# Influence of long-term nutrient deficiency on pollen and anther morphological traits in rye

**DOI:** 10.1093/jxb/eraf537

**Published:** 2025-12-16

**Authors:** Christina Waesch, Noah Gaede, Melanie Wulff, Izdihar Ferhat, Manuela Nagel, Susanne Dunker, Steven Dreissig

**Affiliations:** Leibniz Institute of Plant Genetics and Crop Plant Research (IPK), Seeland, OT Gatersleben, Germany; Institute of Agricultural and Nutritional Sciences, Martin-Luther-University Halle Wittenberg, Halle (Saale), Germany; Leibniz Institute of Plant Genetics and Crop Plant Research (IPK), Seeland, OT Gatersleben, Germany; Institute of Agricultural and Nutritional Sciences, Martin-Luther-University Halle Wittenberg, Halle (Saale), Germany; Institute of Agricultural and Nutritional Sciences, Martin-Luther-University Halle Wittenberg, Halle (Saale), Germany; Institute of Agricultural and Nutritional Sciences, Martin-Luther-University Halle Wittenberg, Halle (Saale), Germany; Ecole Nationale Supérieure de Biotechnologie ENSB, Constantine, Algeria; Leibniz Institute of Plant Genetics and Crop Plant Research (IPK), Seeland, OT Gatersleben, Germany; Helmholtz Centre for Environmental Research (UFZ), Leipzig, Germany; German Centre for Integrative Biodiversity Research (iDiv) Halle-Jena-Leipzig, Leipzig, Germany; Leibniz Institute of Plant Genetics and Crop Plant Research (IPK), Seeland, OT Gatersleben, Germany; Institute of Agricultural and Nutritional Sciences, Martin-Luther-University Halle Wittenberg, Halle (Saale), Germany; German Centre for Integrative Biodiversity Research (iDiv) Halle-Jena-Leipzig, Leipzig, Germany; The University of Adelaide, Australia

**Keywords:** Genotype–environment interaction, GWAS, imaging flow cytometry, male reproductive traits, pollen size, rye, soil nutrient availability, wind pollination

## Abstract

Understanding how environmental factors shape male reproductive traits is crucial for plant breeding and evolutionary biology. Here, we investigated the impact of soil nutrient availability on male reproductive traits in the wind-pollinated grass *Secale cereale*, leveraging the long-term ‘Eternal Rye’ monoculture field trial established in 1878. We analysed 552 rye individuals of a population variety and 736 rye individuals of a diverse rye panel grown under nutrient-deficient and nutrient-enriched conditions. Our results show that nutrient deficiency, compared with nutrient enrichment, significantly reduced anther length and pollen number per anther, whereas pollen size and viability remained stable or increased in the population variety. Under nutrient-rich conditions, we observed a trade-off between pollen size and number, which was absent under nutrient-deficient conditions, suggesting shifts in resource allocation strategies. Importantly, this phenotypic plasticity corresponded to changes in the underlying genetic architecture, with distinct quantitative trait loci (QTLs) detected under nutrient-deficient versus nutrient-enriched environments. These findings highlight the substantial influence of environmental plasticity on male reproductive traits and their genetic control in rye.

## Teaser

Soil nutrient availability shapes male reproductive traits in rye, a wind-pollinated cereal. Environment-specific QTLs and trade-offs highlight strong genotype–environment interactions underlying reproductive plasticity.

## Introduction

The development of viable pollen is a crucial part of the life cycle of plants, ensuring sexual reproduction and seed set. However, male reproductive development, particularly microsporogenesis and microgametogenesis, is extremely sensitive to adverse environmental conditions, whereas the ovary demonstrates greater resilience (Ji *et al.*, 2010; Hedhly, 2011). Stress-induced male sterility caused by drought, cold, or heat reduces seed set in self-pollinating crops such as wheat, barley, and rice, and poses a major threat to crop yields (Dolferus *et al.*, 2011).

Beyond climatic factors, soil nutrient availability also plays a fundamental role in reproductive success such as seed set through female function, but some studies also report on its effect on male reproductive function (Young and Stanton, 1990; Campbell and Halama, 1993; Lau and Stephenson, 1993, 1994). Increased nutrient levels resulted in an increase in size and number of flowers, pollen number, size, and viability, nectar secretion rates, and sugar ratios in animal-pollinated species (Shuel, 1957; Vasek, 1987; Campbell and Halama, 1993; Lau and Stephenson, 1993, 1994; Lau *et al.*, 1995; Muñoz *et al.*, 2005; Burkle and Irwin, 2009; Hoover *et al.*, 2012; Atasay *et al.*, 2013; Vaudo *et al.*, 2022). These traits improve attractiveness to pollinators, offering a selective advantage to plants reliant on animal-mediated pollination.

In contrast, evolution of wind-pollinated species was not driven by pollinator interactions but by traits that maximize pollen output and dispersal. These evolutionary adaptations include producing large quantities of pollen, with a high pollen–ovule ratio, and optimizing pollen aerodynamic features such as a smaller pollen size (20–60 µm), lower mass, and smooth exine sculptures, to reduce settling velocity and facilitate long-distance dispersal (Sapra and Hughes, 1975; Niklas, 1985; Fritz and Lukaszewski, 1989; Ackerman, 2000; Cruden, 2000; Waesch *et al.*, 2025b). Despite this ecological and morphological divergence of wind-pollinated species, relatively little research has explored if soil nutrient availability, particularly under realistic field conditions, influences male reproductive traits in a comparable way, or whether different trade-offs are at play. However, a recent study on grasslands demonstrated that nitrogen fertilization affected pollen quantity and quality, increasing pollen production and allergenicity in grasses, posing a threat to public health (Daelemans *et al.*, 2025). Further on, the genetic architecture underlying the phenotypic plasticity of male reproductive traits in response to environmental variation remains poorly understood. Given the natural variation for male reproductive traits that exists within species, elucidating the genetic basis of this plasticity is critical for both fundamental research and plant breeding, particularly in times of climate change and increasingly variable environments.

Rye (*Secale cereale*, 2*n*=14) is a self-incompatible, wind-pollinated cereal grass belonging to the *Poaceae* family and *Triticeae* tribe. In a recent study, we demonstrated pronounced, heritable within-species variation in pollen size (39–57 µm) and anther length (5.5–14.3 mm) in rye, traits shaped by domestication, and identified several small-effect loci associated with these traits (Waesch *et al.*, 2025b). The genus *Secale* consists of three partially interfertile species: *Secale cereale* (annual), *Secale sylvestre* (annual), and *Secale strictum* (perennial). While *S. sylvestre* and *S. strictum* are exclusively wild species (Frederiksen and Petersen, 1998; Schreiber *et al.*, 2019, 2021; Rabanus-Wallace *et al.*, 2021), *S. cereale* includes domesticated, weedy, feral, and wild populations. Extensive gene flow occurs among these groups due to the cross-pollinating and self-incompatible nature of rye (Schreiber *et al.*, 2019, 2022; Waesch *et al.*, 2024). Rye is best known for its exceptional tolerance against abiotic and biotic stress. It can survive under severe cold and drought conditions, and can thrive in marginal soils and at high altitudes (Geiger and Miedaner, 2009; Li *et al.*, 2011).

The world’s second oldest long-term soil fertilization experiment, known as ‘Eternal Rye’, involves rye monoculture and was established by Julius Kühn in 1878. For a long time, its primary objective was to study the long-term effects of nutrient deficiency, as well as organic and mineral fertilization (nutrient enrichment), on crop yield and soil fertility (Schmidt *et al.*, 2000; https://ltehub.landw.uni-halle.de/eternal-rye/). However, this trial now offers the opportunity to investigate how soil nutrient availability influences male reproductive traits in a wind-pollinator. Due to advances in next-generation sequencing and the recently assembled reference genome of rye (Li *et al.*, 2021; Rabanus-Wallace *et al.*, 2021), it also has the potential to help to understand the underlying genetic architecture of male reproductive traits in response to soil nutrient availability.

In this experimental study, we aimed to build on previous work by asking whether nutrient deficiency reduces anther and pollen size, pollen count per anther, and pollen viability, and how relationships among these traits and their genetic architecture shift under different nutrient conditions in a wind-pollinated cereal. To address these questions, we analysed 552 rye individuals from a population variety and 736 rye individuals from a diversity panel, both grown under nutrient-deficient and nutrient-enriched conditions.

Our results revealed a significant negative effect of nutrient deficiency on anther length and pollen count per anther. In contrast, pollen size and viability were either unaffected or slightly increased under nutrient-deficient conditions. Under nutrient enrichment conditions, we observed a negative correlation between pollen size and pollen count, suggesting a trade-off in resource allocation, which was absent under nutrient deficiency. Additionally, we identified distinct sets of quantitative trait loci (QTLs) for anther length under nutrient deficiency and enrichment, and QTLs for pollen size were detected exclusively under nutrient-deficient conditions. These findings suggest a genotype-by-environment interaction influencing male reproductive traits in response to soil nutrient availability. Our study extends our knowledge about the phenotypic plasticity of male reproductive traits of a wind-pollinator to different soil nutrient availabilities and its underlying genetic architecture.

## Materials and methods

### Plant material and field trials

This study was based on two rye populations: ‘Conduct’, a commercial population variety (hereafter referred to as the population variety), and a rye diversity panel consisting of a mixture of 1000 rye genebank accessions (Waesch *et al.*, 2025a), each represented by five seeds (hereafter referred to as the diversity panel).

The two rye populations were cultivated at the experimental field station in Halle (Saale), Germany (51°29′52.7″N, 11°59′31.3″E), but in different growing seasons: the population variety during the 2021/2022 season, and the diversity panel during 2022/2023. Both populations were grown in two treatments of the long-term field trial ‘Eternal Rye’ including nutrient deficiency and nutrient enrichment treatment. The nutrient deficiency treatment involves a soil plot which remained unfertilized since the establishment of the ‘Eternal Rye’ in 1878, resulting in long-term depletion of both micro- and macronutrients due to continuous rye monoculture (Supplementary Table S1). The nutrient enrichment treatment involves a soil plot with N, P, and K fertilization according to local agricultural practices (60 kg N ha^–1^, 24 kg P ha^–1^, 75 kg K ha^–1^). Both populations were sown in plots of 18 m^2^ with 270 grains m^–2^ and 15 cm row spacing for both treatments.

For pollen and anther phenotyping, 4–6 spikelets were collected per individual from the main tiller, specifically from the centre of the spike before anther dehiscence, with yellow anthers still enclosed within the florets. The spikelets were placed into a 2 ml Eppendorf tube containing 1.5 ml of fixative solution [3:1 ethanol (99%):glacial acetic acid (99%)]. To accurately estimate pollen count per anther, a single mature anther was separately collected before anther dehiscence and stored in a 1.5 ml Eppendorf tube with 1 ml of the same fixative, but only for individuals of the diversity panel. All samples were stored at 4 °C until further processing.

### Phenotyping

In this study, the following phenotypic traits were assessed: grain yield (g m^–2^), plant height (cm), pollen count per anther, anther length (mm), pollen size (µm) measured as pollen length (longest axial dimension), and pollen viability (%).

#### Yield and plant height measurement

Plant height was measured for the diversity panel in centimetres from the ground to the tip of the erect spikes, excluding awns, during flowering. Grain yield was measured in g m^–2^, harvesting all spikes in the 18 m^2^ plots by hand for both populations.

#### Anther length measurement

A total of six anthers were extracted from a single fixed spikelet per individual using tweezers. The anthers were arranged and imaged under a stereo microscope equipped with a camera (Stemi 508, Zeiss; AxioCam ERc 5s, Zeiss). Anther length was measured using the image analysis software ImageJ, and the median length per individual was calculated from the six measurements.

#### Pollen size and fluorescence phenotyping

Pollen size and fluorescence were analysed using multispectral imaging flow cytometry (MIFC) following the protocols from Dunker *et al.* (2021) and Waesch *et al.* (2025b). The samples were processed using the multispectral imaging flow cytometer ImageStream® X MK II (Amnis part of Cytek, Amsterdam, the Netherlands) autosampler, and images were captured at ×20 object magnification with the instrument-specific INSPIRE Software (v.200.1.620.1). Pollen size information was extracted based on images captured by the first camera (488 nm laser excitation) of channel 1 (brightfield image), and pollen autofluorescence traits were extracted based on images of channel 2 (528/65 nm BP filter), channel 3 (577/35 nm BP filter), channel 4 (610/30 nm BP filter), and channel 5 (702/85 nm BP filter). The IDEAS software (v.6.2.187.0) was used to extract high-quality, single pollen images of fertile pollen based on a stepwise gating approach described in Waesch *et al*. (2025b). The data from the high-quality, fertile pollen fraction were converted into FCS file format and analysed in the R environment (version 4.4.1). Only individuals with ≥50 pollen images were included in this analysis. Outliers based on pollen length, defined as the longest axial dimension of the pollen, were excluded if they fell below the first percentile or above the 99th percentile. For each individual, the median values of pollen size were calculated and used for further analysis.

#### Pollen count measurement

Pollen count per anther was assessed for a subset of 237 individuals (nutrient deficient, *n*=115; nutrient enrichment, *n*=122) of the diversity panel using impedance flow cytometry. Based on a fixed single anther sample, pollen was extracted by gently opening the anthers with a pestle, followed by vortexing the samples for 30 s. The resulting suspension was centrifuged at 13 300 *g* for 2 min, and the supernatant was carefully discarded. The pollen pellet was resuspended in 1 ml of measurement buffer (AF11; Amphasys©, Lucerne, Switzerland). To remove cellular debris, the suspension was filtered through a 100 μm mesh filter (CellTrics, Sysmex Partec GmbH, Germany) into a new tube. The original 1.5 ml Eppendorf tubes were then rinsed with an additional 2 ml of measurement buffer, which was also filtered into the same tube. Prior to measurement, the final suspension was vortexed for 30 s. Impedance flow cytometric measurements were conducted using the Ampha Z32 cytometer (Amphasys©) equipped with the D01266 chip (channel size: 120 μm), operating at 2 MHz. Measurements continued until the entire pollen suspension had been analysed. Data acquisition and gating were performed using AmphaSoft version 2.0 (Amphasys©).

#### Pollen viability

Pollen viability was analysed for a subset of 218 genotypes of both the population variety (nutrient deficient, *n*=41; nutrient enrichment, *n*=40) and the diversity panel (nutrient deficient, *n*=50; nutrient enrichment, *n*=87) using acetocarmine, staining chromatin of viable pollen a bright red colour. For each individual, a single anther was extracted from fixed flower samples and transferred to a new 1.5 ml Eppendorf tube with 1 ml of fixative solution [3:1 ethanol (99%):glacial acetic acid (99%)]. The anther was gently opened using a pestle, followed by vortexing for 30 s. Samples were then centrifuged at 13 300 *g* for 2 min, and the supernatant was carefully removed. The resulting pellet was resuspended in 50 µl of acetocarmine solution (1% carmine in 45% acetic acid). Two microscope slides per individual were prepared as technical replicates by using 15 µl of the suspension per slide and covering with a coverslip. For each replicate, 10–15 non-overlapping microscopic images were acquired at ×10 magnification (Axio Scope.A1, AxioCam 105 color; Zeiss).

In order to enable high-throughput analysis of all pollen images, we used the platform Roboflow (https://roboflow.com) to train a deep learning-based object detection model. A total of 355 microscopic images were manually annotated using the segmentation tool in Roboflow. Each pollen grain was enclosed in a bounding box and labelled as either aborted, semi-vital, or normal (Supplementary Fig. S1A). The annotated dataset was divided into training (315 images), validation (23 images), and test (17 images) sets. A YOLOv11 object detection model was trained on this dataset, incorporating data augmentation techniques (e.g. horizontal and vertical flip, 0% minimum and 20% maximum zoom), to enhance model generalization. The final model achieved a mean average precision (mAP) of 90.4%, with a precision of 80% and a recall of 91.3% on the validation set. Following model prediction, the data on the count of annotations per image were exported, and pollen viability per individual was calculated as the mean of both technical replicates using the formula:


$$\mathrm{Pollen}\;\mathrm{viability}=\frac{\mathrm{count}\;\mathrm{of}\;\mathrm{normal}\;\mathrm{pollen}}{\left(\mathrm{count}\;\mathrm{of}\;\mathrm{aborted}\;\mathrm{pollen}+\mathrm{count}\;\mathrm{of}\;\mathrm{normal}\;\mathrm{pollen}\right)}$$

Genotypes with ≥25% of semi-vital pollen were removed from the analysis, as it is unclear whether these pollen grains were not yet fully developed or if other developmental constraints had occurred. To evaluate the accuracy of the object detection model, we compared pollen viability estimates for 233 rye genotypes derived from the object detection model with estimates obtained through manual counting in ImageJ, using pollen image data from the rye population described in Waesch *et al.* (2025b). This revealed a high correlation (*R*=0.93, *P*<0.001; Supplementary Fig. S1B) between the two methods, indicating that the object detection model yields reliable pollen viability estimates. Consequently, this pipeline was applied to the full image dataset of both the population variety and the diversity panel to estimate pollen viability per individual.

### Genotyping by sequencing

We used sequenced data previously published obtained from the European Nucleotide Archive under accessions number PRJEB88192 (Waesch *et al.,* 2025a). In brief, 726 and 552 randomly selected plants of the diversity panel and population variety, respectively, were sampled and sequenced on an Illumina NovaSeq 6000 platform at IPK Gatersleben (118 cycles, single-end reads). Raw data were processed as described in Waesch *et al.* (2025a), resulting in a final set of 51 704 high-quality single nucleotide polymorphisms (SNPs; <10% missing data, >1% minor allele frequency, >4 reads per SNP, <100 reads per SNP, only bi-allelic SNPs).

### Population and quantitative genetic metrices

Population structure was assessed using principal component analysis (PCA) based on a genetic covariance matrix, implemented with the snpgdsPCA() function from the SNPRelate package in R (Zheng *et al.*, 2012). Nucleotide diversity (*π*) per site and the inbreeding coefficient (*F*) were calculated with VCFtools (Danecek *et al.*, 2011). Linkage disequilibrium (LD) decay was estimated as the squared correlation coefficient (*r*^2^) between SNP pairs, considering distances between 1 000 000 bp and 20 000 000 bp using VCFtools. Genome-wide association scans (GWASs) were performed separately for nutrient enrichment and nutrient deficiency treatments using the FarmCPU algorithm (Fixed and random Circulating Probability Unification) implemented in GAPIT V3 (Wang and Zhang, 2021). To validate significant marker–trait associations, 100-fold repeated random subsampling was performed, sampling 95% of the dataset in each iteration.

SNP-based heritability estimates were calculated using the GCTA tool (gcta64), following the method described by Yang *et al.* (2011). The SNP matrix was converted from VCF to PLINK format, and a genetic relationship matrix (GRM) was generated using the -make-grm function. Heritabilities for individual anther and pollen size were calculated using the -grm function.

### Data analysis

All statistical analyses were conducted in the R environment (version 4.4.1). Pearson’s correlation coefficients were calculated using the rcorr() function from the Hmisc package. Group comparisons to assess statistical significance were performed using Wilcoxon test (Mann–Whitney U-test) with the wilcox_test() function in the rstatix package, with Bonferroni correction applied to adjust for multiple testing. A linear regression analysis was performed using the lm() function in the stats package.

## Results

### Population structure

This study investigated a total of 736 individuals of a rye diversity panel and 552 individuals of a rye population variety. Both populations were cultivated under two contrasting soil nutrient conditions: one with severe nutrient deficiency, and the other with mineral fertilization serving as the nutrient enrichment treatment.

**Fig. 1.**
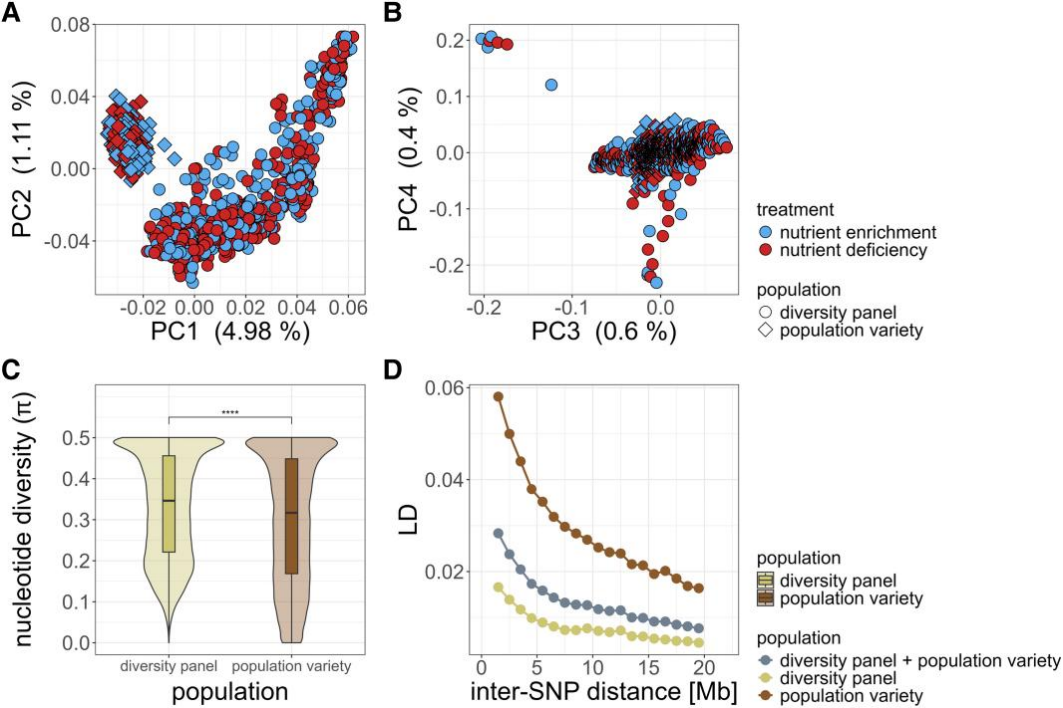
Evaluation of population structure, genetic diversity, and linkage disequilibrium (LD) in the rye population variety and rye diversity panel using 51 704 SNPs. (A) Principal component analysis (PCA) of PC1 versus PC2 and (B) PC3 versus PC4, with colours indicating soil nutrient treatments (red, nutrient deficiency; blue, nutrient enrichment) and symbols representing populations (circles, diversity panel; diamonds, population variety). (C) Nucleotide diversity analysis demonstrates significant higher genetic diversity in the diversity panel (ochre) compared with the population variety (brown), based on a Mann–Whitney U-test; ****P<0.0001. (D) LD decay across a 1–20 Mb genomic window shows higher LD in the population variety, lower LD in the diversity panel, and intermediate levels in the combined population (grey).

Based on a PCA, we analysed the population structure of our two populations using 51 704 SNPs, which revealed distinct clustering patterns between the two populations (Fig. 1A). The population variety forms a separate cluster from the diversity panel, displaying the lowest overall PC1 values, whereas the genotypes of the diversity panel form as a gradient along PC1 from high to low, reflecting a degree of genetic differentiation from wild and feral up to domesticated rye (Schreiber *et al.*, 2022; Waesch *et al.*, 2025b). Overall, population structure was low, as the first two principal components together explained only 6.09% of the total genetic variation.

Regarding PC3 and PC4, a few individuals from the diversity panel separated from the main cluster (Fig 1B; Supplementary Fig. S2A). To clarify the origin of these outlier lines, we performed an identity-by-state (IBS) analysis using the genetic dataset of Schreiber *et al*. (2022), which includes 266 rye genebank accessions. For each outlier, we identified the top two rye lines with the highest IBS values. This analysis suggested that individuals with high PC4 values are likely to be weedy *S. cereale*, whereas those with negative PC4 values most probably represent domesticated *S. cereale* (Supplementary Fig. S2B). A joint PCA of our dataset and that of Schreiber *et al*. (2022) confirmed this classification: the likely domesticated lines clustered at lower PC1 values within the domesticated range, while the likely weedy lines clustered at higher PC1 values within the feral and wild range. This result also excluded the possibility that these lines belong to *S. sylvestre* or *S. strictum* (Supplementary Fig. S2C). Together, these analyses indicate that our rye diversity panel consists exclusively of *S. cereale* individuals, encompassing feral, wild, weedy, and domesticated origins.

Importantly, no separation was observed between the nutrient deficiency and nutrient enrichment treatments within either population, indicating that the treatments represent similar genetic diversity, which was further confirmed by analysis of nucleotide diversity, revealing no significant difference between the treatments within the populations (Supplementary Fig. S2E, F). As expected, we observed a higher nucleotide diversity in the diversity panel compared with the population variety (Fig. 1C). We also observe a higher LD in the population variety compared with the diversity panel (Fig. 1D).

As ploidy is known to influence traits such as pollen size, we aimed to eliminate this confounding effect from our analysis. Based on analysis of the inbreeding coefficient (*F*) and comparison with reference diploid and tetraploid samples from Waesch *et al.* (2025b) (Supplementary Fig. S2D), we identified 66 genotypes within the diversity panel as likely tetraploids. These genotypes were excluded from subsequent analyses.

### Soil nutrient deficiency negatively affects anther length and pollen count but not pollen size and viability

**Fig. 2.**
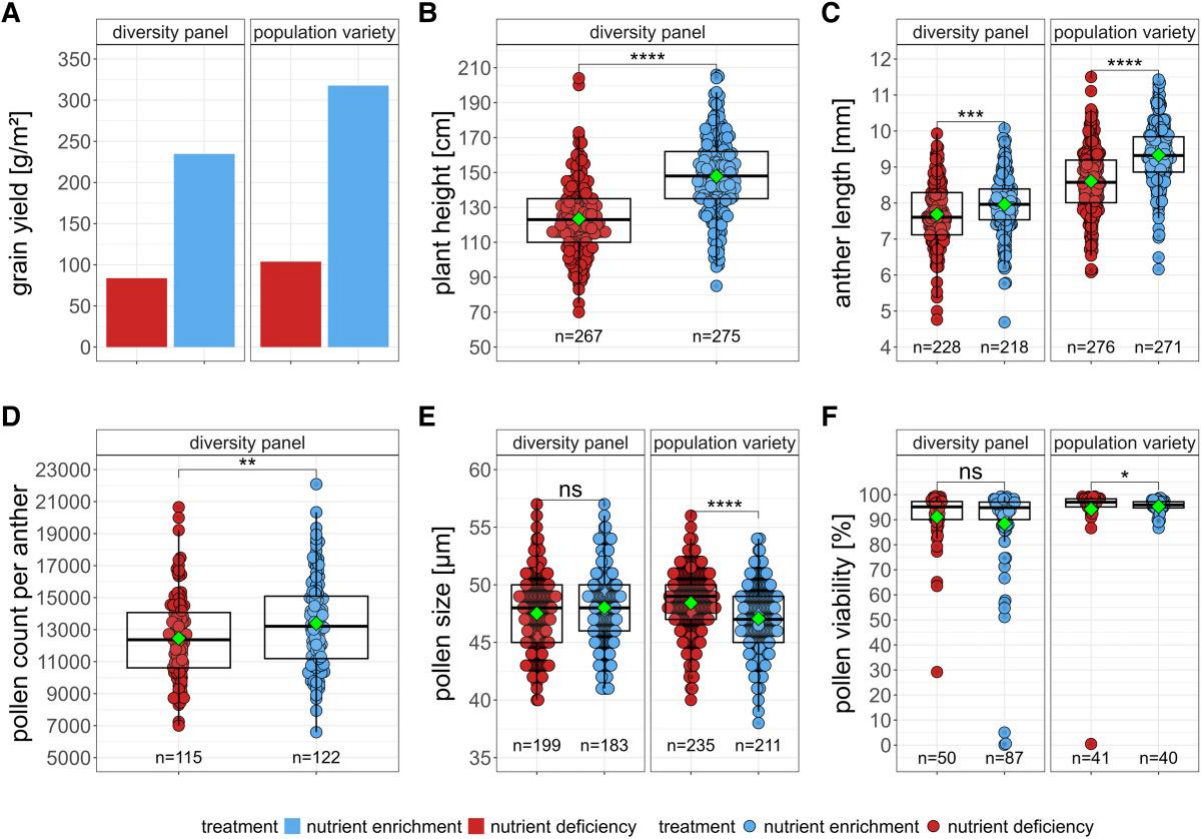
Impact of long-term nutrient deficiency on vegetative and male reproductive traits in rye. (A) Reduction of grain yield by >60% due to nutrient deficiency for both rye populations. (B) Plant height was significantly reduced by nutrient deficiency in the diversity panel. (C) Nutrient deficiency causes significant reduction in anther length for both populations. (D) Pollen count per anther was significantly reduced due to nutrient deficiency in the diversity panel. (E) Pollen size and (F) pollen viability were either not affected or significantly increased due to nutrient deficiency in the diversity panel and the population variety, respectively. Mann–Whitney U-test with Bonferroni correction applied; ns=not significant, *P<0.05, **P<0.01, ***P<0.001, ****P<0.0001; colour code represents soil nutrient treatment (blue, nutrient enrichment; red, nutrient deficiency), and green diamonds indicate mean values.

Generally, it is known that mineral fertilization increases grain yield and plant biomass (Frink *et al.*, 1999; Tilman *et al.*, 2002; Feng *et al.*, 2023). In our study, nutrient deficiency led to a substantial reduction in grain yield, with decreases of 64–67% observed in both rye populations (Fig. 2A). We measured plant height in the diversity panel at the individual level, given the greater variation observed in this trait compared with the population variety. Nutrient deficiency had a significant negative effect on plant height, with stressed individuals being on average 14.2% shorter (−21.3 cm) than those in nutrient enrichment plots (Fig. 2B; Supplementary Table S2). These findings confirm the strong difference in the soil nutrient availability between the nutrient deficiency and nutrient enrichment plots.

Regarding male reproductive traits, reduced soil nutrient availability significantly decreased anther length by on average 3.8% (−0.38 mm) in the diversity panel and by on average 7.5% (−0.7 mm) in the population variety (Fig. 2C; Supplementary Table S2). Pollen count per anther, measured exclusively in the diversity panel, was also significantly reduced under nutrient deficiency, with an average decrease of 6.36% (−839 pollen grains) (Fig. 2D; Supplementary Table S2). Interestingly, all measures of pollen autofluorescence were significantly diminished by nutrient deficiency across both rye populations (Supplementary Fig. S3A–D; Supplementary Table S2).

In contrast, pollen size and viability were not negatively affected by the nutrient deficiency (Fig. 2E, F; Supplementary Table S2). No significant differences were detected between treatments for these traits in the diversity panel. Notably, in the population variety, nutrient deficiency was associated with a significant increase in both pollen size (+2.8%, +1.3 µm) and pollen viability (+1.2%).

Our findings highlight the dependence of male reproductive traits such as anther length and pollen count on soil nutrient availability, similar to other biomass traits such as yield and plant height. In contrast, pollen size and viability appear to be buffered against soil nutrient limitation, indicating their importance for reproductive assurance.

### Nutrient availability modulates reproductive trait correlations and trade-offs

**Fig. 3.**
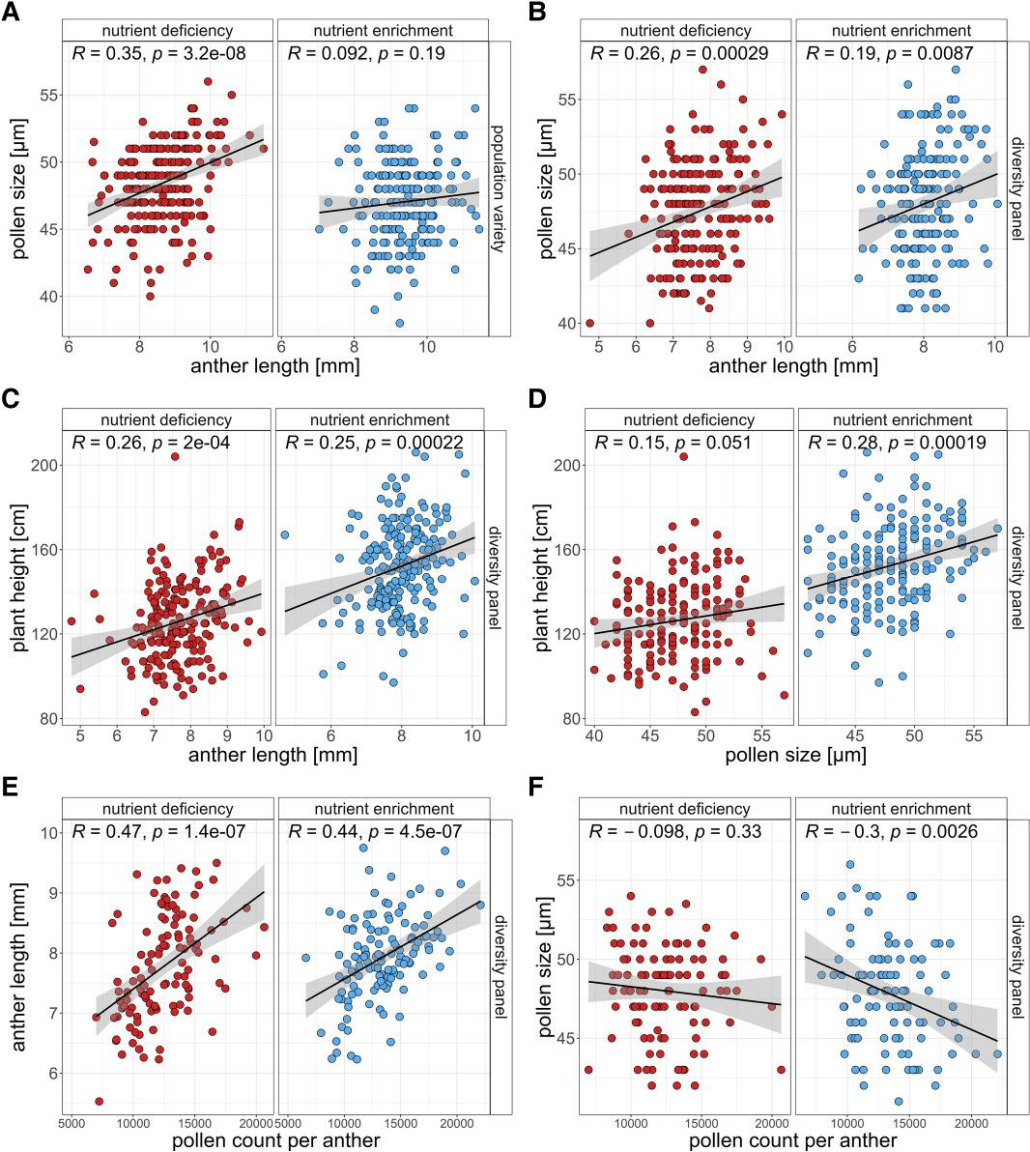
Soil nutrient availability influences relationships among male reproductive traits in both populations. Correlation scatterplots display: (A, B) anther length versus pollen size in the population variety and diversity panel; (C, D) plant height versus anther length and pollen size in the diversity panel; and (E, F) anther length versus pollen count per anther and pollen size versus pollen count per anther in the diversity panel. Pearson correlation coefficients with corresponding *P*-values are shown; colour code represents soil nutrient treatment (blue, nutrient enrichment; red, nutrient deficiency).

In a previous study, we reported a modest positive correlation between pollen size and anther length in a diverse rye panel (*R*=0.32, *P*<0.001; Waesch *et al.*, 2025b). In the present study, we confirmed this relationship, with a slightly stronger correlation under nutrient deficiency (*R*=0.26–0.35, *P*<0.001) than under nutrient enrichment conditions (*R*=0.092–0.22, *P*>0.05 to *P*<0.01) (Fig. 3A, B). Notably, this correlation was absent in the nutrient-enriched conditions of the population variety (Fig. 3A).

A small positive correlation was observed between plant height and anther length in the diversity panel under both treatments (*R*=0.2–0.27, *P*<0.05 to *P*<0.001) (Fig. 3C). In contrast, no significant relationship was observed between pollen size and plant height under either condition (*R*=0.18–0.19, *P*>0.05) (Fig. 3D).

After removing outlier genotypes with low pollen viability (≤ 70%), potentially due to genetic factors causing male sterility, we detected a positive correlation between anther length and pollen viability (*R*=0.33, *P*<0.01) in the diversity panel under nutrient-enriched conditions. In contrast, no correlation was observed under nutrient-deficient conditions (Supplementary Fig. S4A). Pollen size and pollen viability displayed positive correlations in the diversity panel under both nutrient enrichment (*R*=0.25, *P*<0.05) and nutrient deficiency conditions (*R*=0.46, *P*<0.01), whereas in the population variety this positive association was only found under nutrient enrichment conditions (*R*=0.38, *P*<0.05) (Supplementary Fig. S4B). This would indicate that pollen size is interlinked with pollen fertility.

Previous research has demonstrated a positive association between anther length and pollen count per anther in various crops (Oka and Morishima, 1967; Milohnic and Jost, 1970; De Vries, 1974; Pickett, 1993; Nguyen *et al.*, 2015; Severova *et al.*, 2022). Consistent with these findings, we observed a significant positive correlation under both nutrient enrichment and nutrient deficiency conditions (*R*=0.44–0.45, *P*<0.001) (Fig. 3E), suggesting robust developmental constraints between these traits regardless of soil nutrient availability. Interestingly, a significant negative correlation between pollen size and pollen count was found under nutrient enrichment conditions (*R*= −0.3, *P*<0.001), whereas no such relationship was observed under nutrient deficiency (*R*= −0.078, *P*>0.05) (Fig. 3F). The negative correlation under nutrient enrichment could indicate a resource allocation strategy, where plants either produce many pollen but smaller in size, whereas others invest resources into producing larger pollen but fewer of them. Ultimately, this results in a pollen size–number trade-off. This trade-off is absent under nutrient deficiency, probably since plants prioritize investing resources into pollen size, to maintain high fertility.

### Distinct genetic architecture of pollen and anther length in response to nutrient availability

Intrigued by the phenotypic plasticity observed under the distinct soil nutrient treatments, we asked if these variations were heritable and if the genetic architecture was shaped by the environmental plasticity. For this purpose, we calculated narrow-sense heritability for anther and pollen size, as both traits were assessed across >200 genotypes. We found a high heritability for anther length under nutrient enrichment conditions (*h*^2^=0.85, SE=0.11) (Supplementary Table S3), consistent with previous findings (Waesch *et al.*, 2025b). However, this value decreased under nutrient deficiency (*h*^2^=0.5, SE=0.11), suggesting that environmental factors exert a stronger influence on anther length in stressed conditions. In contrast, pollen size displayed a different pattern. Under nutrient enrichment conditions, we found a low heritability (*h*^2^=0.26, SE=0.14), aligning with earlier results (Waesch *et al.*, 2025b). Interestingly, heritability increased under nutrient deficiency (*h*^2^=0.49, SE=0.15), implying a stronger genetic influence on this trait in a nutrient stress environment. This could indicate that specific genetic factors become more influential under nutrient deficiency, while under nutrient enrichment conditions this trait may be governed by many small-effect loci.

Next, we performed GWAS for pollen and anther length by combining data of the population variety and the diversity panel. We employed a repeated random subsampling approach, performing 100 GWAS runs in which 95% of the original dataset was selected in each iteration. To minimize false-positive associations, we considered only QTLs with a detection rate >5%, meaning QTLs detected in at least five independent runs.

**Fig. 4.**
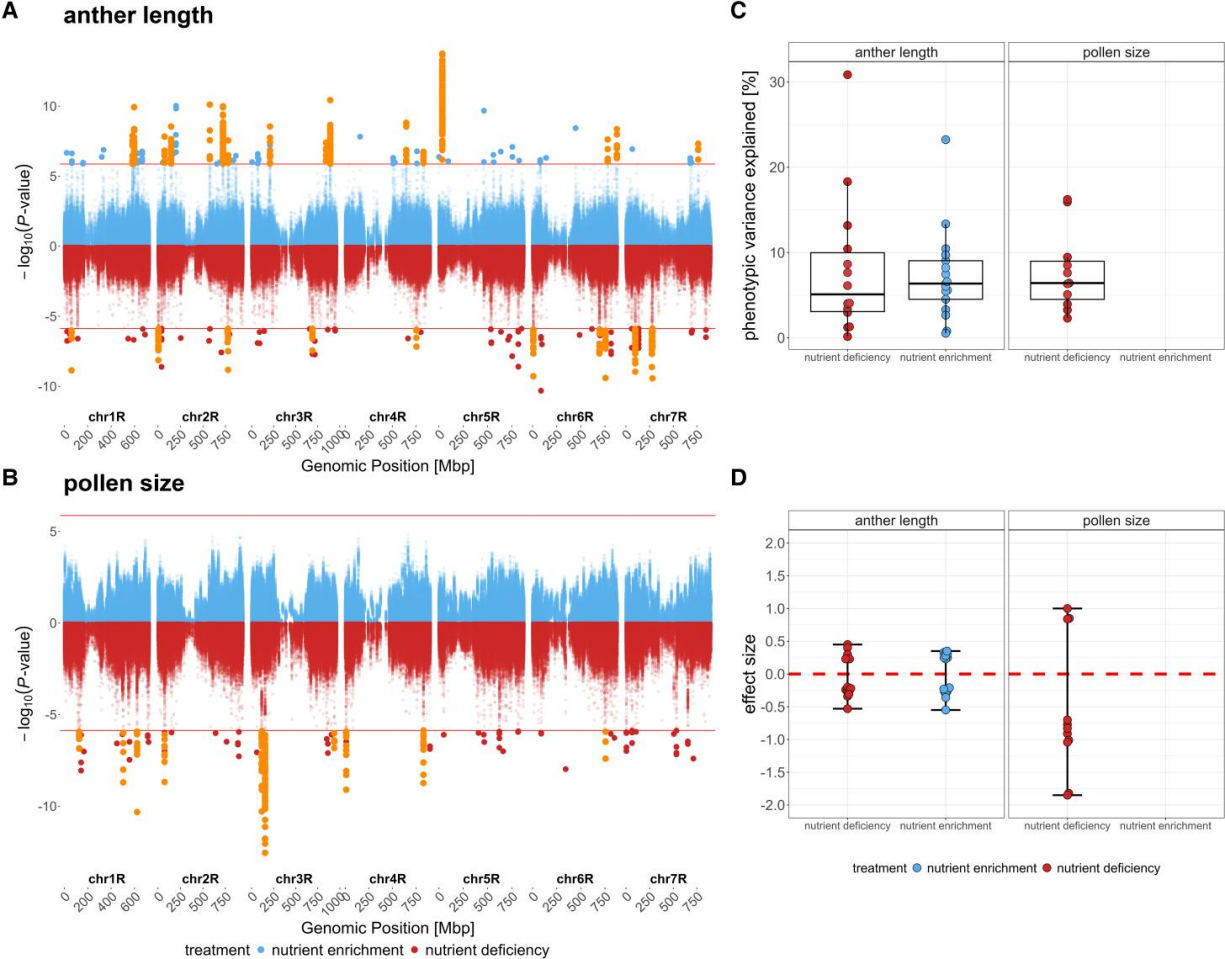
Genetic architecture of pollen and anther length in response to soil nutrient treatment. Manhattan plots from 100 FarmCPU GWAS runs using 95% random subsampling show associations for anther length (A) and pollen size (B) under nutrient enrichment (blue) and nutrient deficiency (red) conditions. *P*-values are displayed on a −log_10_ scale; orange points mark QTLs detected in ≥5% of runs. The red horizontal line indicates the genome-wide significance threshold (0.05/36871). (C) Average phenotypic variance explained for anther (nutrient deficiency *n*=14, nutrient enrichment *n*=17) and pollen size QTLs (nutrient deficiency *n*=11) with detection rate ≥5%. (D) Average effect size of anther (nutrient deficiency *n*=14, nutrient enrichment *n*=17) and pollen size QTLs (nutrient deficiency *n*=11) with detection rate ≥5%.

This analysis identified 17 and 14 QTLs for anther length under nutrient enrichment and nutrient deficiency conditions, respectively (Fig. 4A; Supplementary Table S4). Notably, the QTLs detected under the two treatments did not overlap in genomic position and were not located within ±5 Mb of each other, suggesting distinct, environment-specific genetic regulation of anther length in response to nutrient availability. For pollen size, we detected 11 QTLs under nutrient deficiency conditions, whereas no significant associations were found under nutrient enrichment conditions (Fig. 4B; Supplementary Table S3). Overall, the detected QTLs exhibited low explained variances (Fig. 4C) and small effect sizes (Fig. 4D), consistent with previous findings (Waesch *et al.*, 2025b). Notably, this pattern remained unchanged even under nutrient deficiency conditions. Together, these results demonstrate that the genetic architecture of anther and pollen size is highly influenced by the environmental plasticity.

## Discussion

In this study, we evaluated the impact of soil nutrient availability on male reproductive traits in the wind-pollinated grass *S. cereale*, using the historic ‘Eternal Rye’ soil nutrition experiment established in 1878. We examined both a population variety, representing contemporary breeding material, and a rye diversity panel composed of a mixture of 1000 diverse genebank accessions, including domesticated, weedy, and feral rye. This dual-population approach was motivated by evidence that domestication-related selection has influenced male reproductive traits in rye (Waesch *et al.*, 2025b). Our results show that nutrient deficiency, compared with nutrient enrichment, significantly reduced anther length and the number of pollen grains per anther, while pollen size and viability remained stable or even increased in the population variety. Under nutrient-enriched conditions, we observed a trade-off between pollen size and number, but this relationship was absent under nutrient-deficient conditions, suggesting a shift in resource allocation strategies. Notably, this phenotypic plasticity in pollen and anther traits was translated into changes in their underlying genetic architecture. We detected distinct genetic associations under nutrient-deficient versus nutrient-enriched environments, indicating a strong influence on those traits by the environmental plasticity.

### Pollination mode can influence how soil nutrient availability affects male reproductive traits

In animal-pollinated plants, soil nutrient availability, particularly of nitrogen and phosphorus, has been shown to influence reproductive traits such as flower production, nectar secretion and composition, anther size, and pollen traits (e.g. size, number, composition, and viability). Higher soil nutrient levels increased these traits, ultimately influencing plant–pollinator interactions (Vasek, 1987; Lau and Stephenson, 1993, 1994; Lau *et al.*, 1995; Burkle and Irwin, 2009; Hoover *et al.*, 2012; Atasay *et al.*, 2013; Ceulemans *et al.*, 2017; Vaudo *et al.*, 2022; Gao *et al.*, 2023). For animal-pollinators, investment in such traits improves attractiveness to pollinators and confers a selective advantage. In contrast, wind-pollinated species rely on abiotic factors for pollination enforcing different selective pressures. We therefore expected that nutrient availability might affect these traits differently in wind-pollinated plants, reflecting distinct resource allocation strategies.

In rye, anther length and pollen count responded to soil nutrient availability in a pattern similar to that observed in animal-pollinated species, increasing under mineral fertilization and decreasing under nutrient-deficient conditions (Fig. 2C, D). Wind-pollinators benefit from high pollen production to maximize chances of successful fertilization (Cruden, 2000). Therefore, the observed increase in anther length and pollen count under nutrient enrichment is likely to reflect an adaptive allocation strategy to increase pollen output. However, under nutrient-limited conditions, although both traits were reduced, the reduction remained modest at <10% (Fig. 2C, D), compared with a >60% drop in grain yield (Fig. 2A). This suggests, that even under resource limitations, rye maintains a baseline investment in these traits to ensure sufficient pollen production for effective pollination which ultimately leads to seed set.

In contrast, pollen size and viability remained stable, or even increased in the population variety, under nutrient deficiency, showing no consistent correlation with increased nutrient availability (Fig. 2E, F). This contrasts with animal-pollinated species, where mineral fertilization often leads to increased pollen size (Lau and Stephenson, 1993, 1994; Lau *et al.*, 1995). Next to pollen output, wind-pollinators also optimize pollen aerodynamic features such as a smaller pollen size (20–60 µm) and mass, as well as smooth exine sculptures, all of which reduce settling velocity and promote long-distance dispersal (Sapra and Hughes, 1975; Niklas, 1985; Fritz and Lukaszewski, 1989; Ackerman, 2000; Cruden, 2000; Waesch *et al.*, 2025b). From this perspective, it is logical that there might be a constraint explaining why rye does not invest in increased pollen size with higher soil nutrient availability, as larger pollen grains would hinder dispersal efficiency. However, pollen size is also correlated with nutrient reserves essential for post-pollination processes such as rapid germination and pollen tube growth, whereby larger pollen outperform smaller ones in siring success due to a higher amount of energy reserves (Baker and Baker, 1979; Cruzan, 1990; Manicacci and Barrett, 1995; McCallum and Chang, 2016). Based on that, in wind-pollinated grasses such as rye, there must be a balance for pollen size even under unfavourable conditions to maintain fertility and to ensure proper dispersal.

We also observed a moderate positive correlation between pollen size and pollen viability (Supplementary Fig. S4). However, we used a staining technique to assess viability, which discriminates between aborted and non-aborted, normally developed pollen, thus providing only an indication of the potential of the pollen to germinate. *In vitro* germination assays, measuring germination capacity, pollen tube length, and growth speed, could deliver a more accurate viability estimate and yield more detailed insight into the fertility implications of pollen size. Additionally, previous studies have shown a positive relationship between pollen size and style length, suggesting that longer styles require more energy for successful pollen tube growth and fertilization (Cruden and Miller-Ward, 1981; Plitmann and Levin, 1983; Cruden, 2009). A future study investigating variation in style length or area across diverse rye lines could provide valuable insights into its relationship with pollen size.

Soil nutrient availability can also affect the chemical composition of pollen (Lau and Stephenson, 1994). Previously we demonstrated within-species variation of pollen autofluorescence intensities in rye, which might be linked to biochemical differences (Waesch *et al.*, 2025b). However, pollen autofluorescence was also observed to vary spatiotemporally within a species (Walther *et al.*, 2025). Here, we showed that pollen autofluorescence intensities are significantly increased in response to an increase in soil nutrient availability (Supplementary Fig. S3). Potentially, under nutrient-enriched conditions, more compounds can be accumulated in the pollen, increasing its fluorescence intensity. Such compounds may include sporopollenin as well as various components of the intine and exine, such as proteins, flavonoids, phenolic compounds, and carotenoids, all of which are known to exhibit autofluorescence (Willemse, 1972; Rozema *et al.*, 2001; Roshchina, 2003; Pöhlker *et al.*, 2012; Xue *et al.*, 2020).

Our results indicate that soil nutrient availability in rye not only influences pollen quantity but may also alter its biochemical composition, potentially affecting pollen function and overall male reproductive success.

### Soil nutrient availability modulates pollen size–number trade-off

A central theory of plant reproductive evolution suggests a negative correlation (trade-off) between the number of reproductive modules and the investment per module (Burd, 1999). This commonly manifests in a trade-off between the number of pollen produced per anther and the size of the pollen (Vonhof and Harder, 1995). Negative correlations between pollen count and pollen size were reported for various plant species, indicating a trade-off in resource allocation (Vonhof and Harder, 1995; Sarkissian and Harder, 2001; Yang and Guo, 2004; Hao *et al.*, 2020). We could confirm this negative relationship between pollen size and pollen count per anther for rye lines grown under nutrient-enriched conditions (Fig. 3F). However, under nutrient deficiency conditions, this correlation was absent (Fig. 3F), indicating that the resource allocation strategy is shifted due to the changed soil nutrient environment. Given that pollen size is potentially associated with fertility, as discussed previously, it is plausible that under nutrient-limited conditions, rye reallocates resources to maintain pollen size at the expense of pollen quantity, prioritizing reproductive success under stress.

The assumption of a trade-off between number of reproductive modules and the investment per module could explain why grain yield was decreased by >60% (Fig. 2A), whereas anther size and pollen number per anther were reduced by <10% (Fig. 2C, D) under nutrient-limited conditions. When nutrients are scarce, plants typically maintain the developmental integrity of individual flowers, including anther and pollen traits, while markedly reducing the number of spikelets they initiate or bring to maturity (Morgan, 1993). This shift in resource allocation substantially lowers spikelet number, thereby limiting seed set and ultimately reducing grain yield.

### Environmental consequences of increased pollen output due to mineral fertilization

Numerous studies have demonstrated a strong positive correlation between anther length and pollen count per anther (Oka and Morishima, 1967; Milohnic and Jost, 1970; De Vries, 1974; Pickett, 1993; Nguyen *et al.*, 2015; Severova *et al.*, 2022). While our results confirmed this relationship under both nutrient-limited and nutrient-enriched conditions, the observed correlation was comparatively lower (*R*=0.44–0.45) (Fig. 3E). This discrepancy may stem from differences in sampling design. Instead of measuring multiple biological replicates per genotype, we selected a subset of individuals per population representing a range of anther sizes (small, medium, and large). Mature anthers were collected prior to dehiscence and placed in tubes containing fixative to minimize pollen loss. Unlike previous studies that used counting chambers and extrapolated from a subset of the entire volume (De Vries, 1974), we quantified the entire sample, resulting in a realistic pollen count range of ∼6000–22 000 grains, with an average of ∼13 000 pollen per anther under nutrient enrichment conditions. Linear regression analysis revealed that each 1 mm increase in anther length corresponded to an increase of ∼1792 pollen grains under nutrient enrichment conditions and ∼1443 grains under nutrient stress conditions (Supplementary Fig. S5). These values align closely with the general trend of ∼1000 pollen grains per 1 mm increase in anther length, which was proposed for several wind-pollinated grasses (Severova *et al.*, 2022). Future studies incorporating biological replicates per genotype may help reduce technical variability and further strengthen this relationship. Nonetheless, the consistently high correlation observed across both soil environments suggests robust developmental constraints linking pollen count and anther length.

Because longer anthers produce more pollen, increases in anther length due to mineral fertilization probably heighten pollen release and the associated allergenic burden. A recent study in grasslands demonstrated that nitrogen fertilization increases both pollen production and allergenicity in grasses, posing a potential threat to public health (Daelemans *et al*., 2025). In our experiment, we similarly observed that soil nutrient enrichment led to increased anther length and pollen counts (Fig. 2C, D). Since standard agricultural practices rely on mineral fertilization to maximize grain yields, this implies that rye cultivation could also produce higher pollen outputs, further exacerbating public health risks. Rye pollen is already a major allergen and represents a significant burden each year for human individuals with pollen allergies (D’Amato *et al.*, 2007; García-Mozo, 2017). However, pollen dispersal distance is strongly influenced by plant height, as taller plants release pollen at higher wind speeds, enabling greater dispersal (Niklas, 1985). Winter rye is among the tallest grown cereals in Germany (Laidig *et al.*, 2021); therefore, reducing plant height in rye could be a potential strategy to limit pollen dispersal and mitigate allergenic exposure. In our diversity panel, we found a small positive correlation between plant height and anther length (Fig. 3C). This finding is consistent with evidence from wheat, where plant height has been shown to be positively associated with pollen count per anther, as well as anther and filament length (Beri and Anand, 1971). Moreover, it has been hypothesized that dwarfing genes may negatively affect traits involved in pollen shedding in wheat (Langer *et al.*, 2014). Recently, the gibberellin (GA)-sensitive dwarfing gene *Ddw1* have been introduced to rye hybrid breeding programmes, effectively reducing plant height in rye (Braun *et al.*, 2019). It would be valuable to analyse male reproductive traits in these semi-dwarf lines to determine whether reducing plant height could effectively decrease pollen dispersal and ultimately lower the allergenic burden of rye pollen.

### Underlying genetic architecture of male reproductive traits in response to nutrient availability

Previous studies have characterized the polygenic architecture of anther and pollen traits in different plants, identifying various small-effect loci (Song *et al.*, 2018; Tsuchimatsu *et al.*, 2020; Aguilar-Benitez *et al.*, 2022). However, these studies did not address the genetic basis of genotype-by-environment interactions underlying these traits. Here, we could show that distinct sets of non-overlapping QTLs were detected in response to nutrient limitation and enrichment for anther length, indicating a different genetic response within each environment. Interestingly, our detected QTLs did not overlap with QTLs we detected for pollen and anther length in a previous study (Waesch *et al.*, 2025b). However, the earlier study analysed a different rye population, representing a distinct allelic spectrum, under a different soil nutrient environment. Despite these differences, both studies underscore the polygenic architecture of pollen and anther morphology, with multiple small-effect loci contributing to the phenotypic trait variation.

The QTL with the highest explained variance of 30% detected for anther length under nutrient deficiency was found on chromosome 6R. A candidate gene closest to the QTL was SECCE6Rv1G0422870 which has high similarity to IAA5 (AT1G15580, 78.1% amino acid similarity) in Arabidopsis, which is a transcriptional repressor of auxin signalling. Generally, young anthers during stamen development showed high levels of the auxin indole-3-acetic acid (IAA) in Arabidopsis (Aloni *et al.*, 2006). Auxin is a key phytohormone regulating plant growth and development, and its signalling genes are expected to play a role in adaptation of the plant to environmental conditions (Mockaitis and Estelle, 2008; Lavy and Estelle, 2016; Leyser, 2018). Taken together, these findings suggest that auxin signalling components near this QTL may contribute to the modulation of anther development under nutrient-limited conditions, potentially representing a key mechanism in stress adaptation.

Another QTL showing high explained variance detected for anther length under nutrient enrichment conditions is located on chromosome 5R, and its candidate gene SECCE5Rv1G0301950 encodes a potential remorin protein. An orthologue was detected in rice GSD1 (Grain Setting Defect 1; 87.5% amino acid similarity) which encodes a remorin protein that is localized to the plasma membrane and plasmodesmata of phloem companion cells. It influences grain setting by regulating the transport of carbohydrates from photosynthetic tissues to the phloem (Gui *et al.*, 2014). Similarly, the remorin protein of rye could affect transport of photoassimilates within anthers and thereby affect anther size.

For pollen size, we detected QTLs only under nutrient deficiency conditions. This finding may explain the observed increase in heritability and lack of reduction in pollen size in response to nutrient deficiency. It suggests that, under nutrient deficiency conditions, a distinct subset of genetic factors becomes active to preserve pollen size, maintaining male fertility, whereas under nutrient enrichment conditions the trait appears to be under polygenic control.

## Conclusion

Taken together, our results revealed distinct patterns of phenotypic plasticity of male reproductive traits in response to soil nutrient availability in a wind-pollinated grass. We demonstrated that anther length and pollen count were negatively affected by reduction of soil nutrient availability, whereas the opposite was shown for pollen size and viability, which is in contrast to results found in animal-pollinators. The soil environment also modulated the relationships among the male reproductive traits and resulted in some cases in a changed resource allocation strategy. We found distinct sets of genetic associations for anther and pollen size in response to the two soil nutrient conditions. This highlights that genotype-by-environment interactions influence male reproductive traits in response to soil nutrient availability. Our study extends the knowledge about the phenotypic plasticity of male reproductive traits in response to soil nutrient environments.

## Supplementary Material

eraf537_Supplementary_Data

## Data Availability

Raw sequence data of this study are available at the European Nucleotide Archive under accession number PRJEB88192. Genotypes of the diversity panel and population variety with corresponding phenotypic traits are provided in Supplementary Table S1.

## Supplementary data

The following supplementary data are available at *JXB* online.

Table S1. Level of soil micro- and macronutrients in nutrient enrichment and deficiency treatment based on Waesch *et al*. (2025a).

Table S2. Phenotypic information of all rye genotypes used in this study.

Table S3. Heritability estimates for anther and pollen length for both populations under nutrient enrichment, nutrient deficiency, or combined.

Table S4. Description of QTLs with a detection rate ≥5% detected in 100-fold GWAS after applying a repeated random subsampling.

Fig. S1. Roboflow-based approach to analyse rye pollen image data.

Fig. S2. Identification of origin of rye lines of the diversity panel clustering in PC3–PC4.

Fig. S3. Pollen fluorescence intensity is significantly higher under nutrient-enriched conditions for both rye populations.

Fig. S4. Correlations of pollen viability with anther length and pollen size under nutrient deficiency and enrichment in a rye diversity panel and a population variety.

Fig. S5. Linear regression model for anther length and pollen count in the diversity panel for nutrient enrichment and nutrient deficiency treatment.
