# Supplementary material for: Influence of long-term nutrient deficiency on pollen and anther morphological traits in rye
Source: J Exp Bot. 2025 Dec 16;77(6):1903–16. doi: 10.1093/jxb/eraf537 (PMC13016982; doi:10.1093/jxb/eraf537)
Supplement: eraf537_Supplementary_Data [file eraf537_supplementary_data.zip › jexbot316836-file001.pdf]

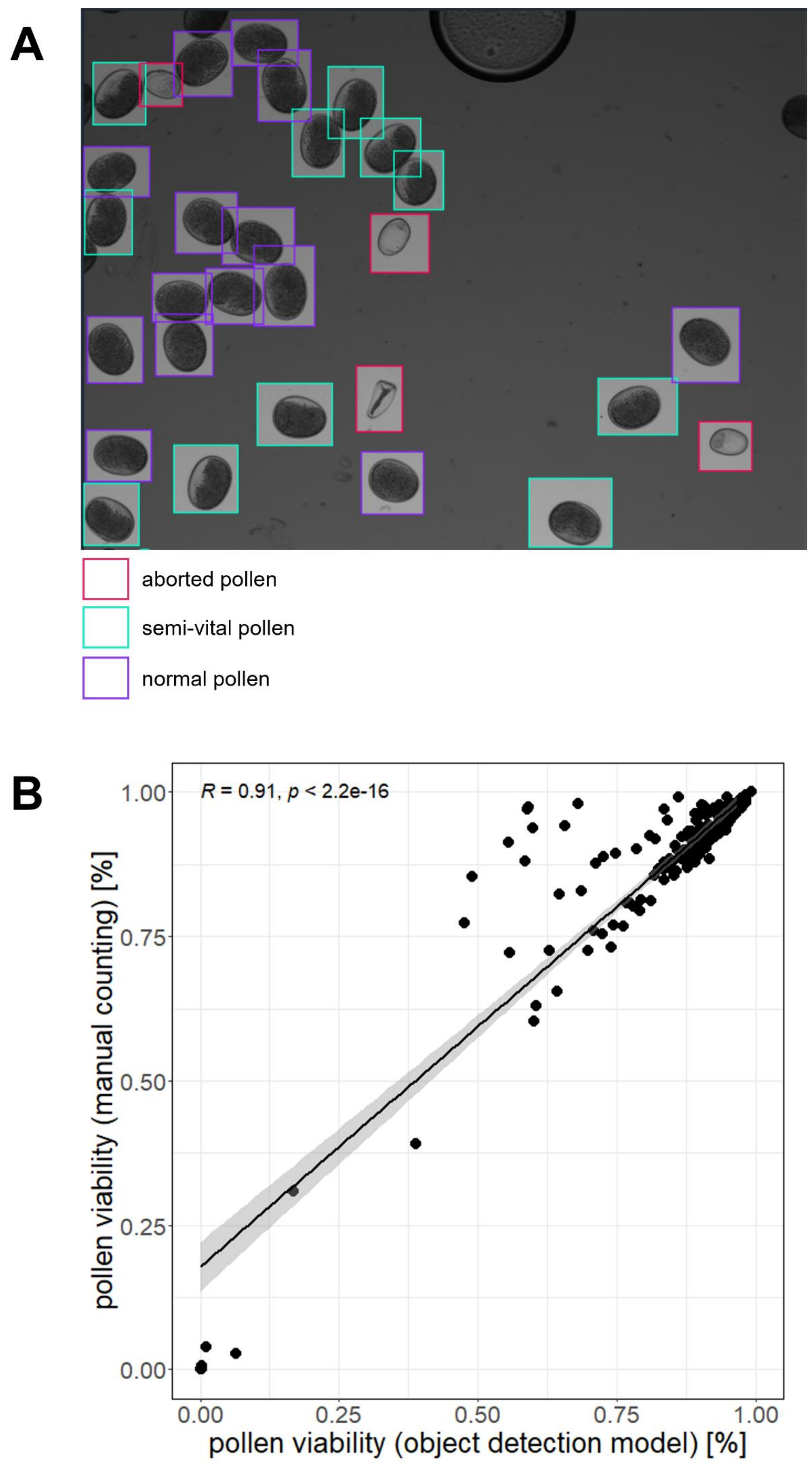

Supplementary Fig. S1: Roboflow-based approach to analyse rye pollen image data. **(A)** Example image of three pollen annotation categories of aborted pollen (red), normal pollen (purple) and semi-vital pollen (green). **(B)** Scatter Plot comparing pollen viability data of 233 rye genotypes pollen viability estimates of manual counting and using the trained Roboflow-based object-detection model.

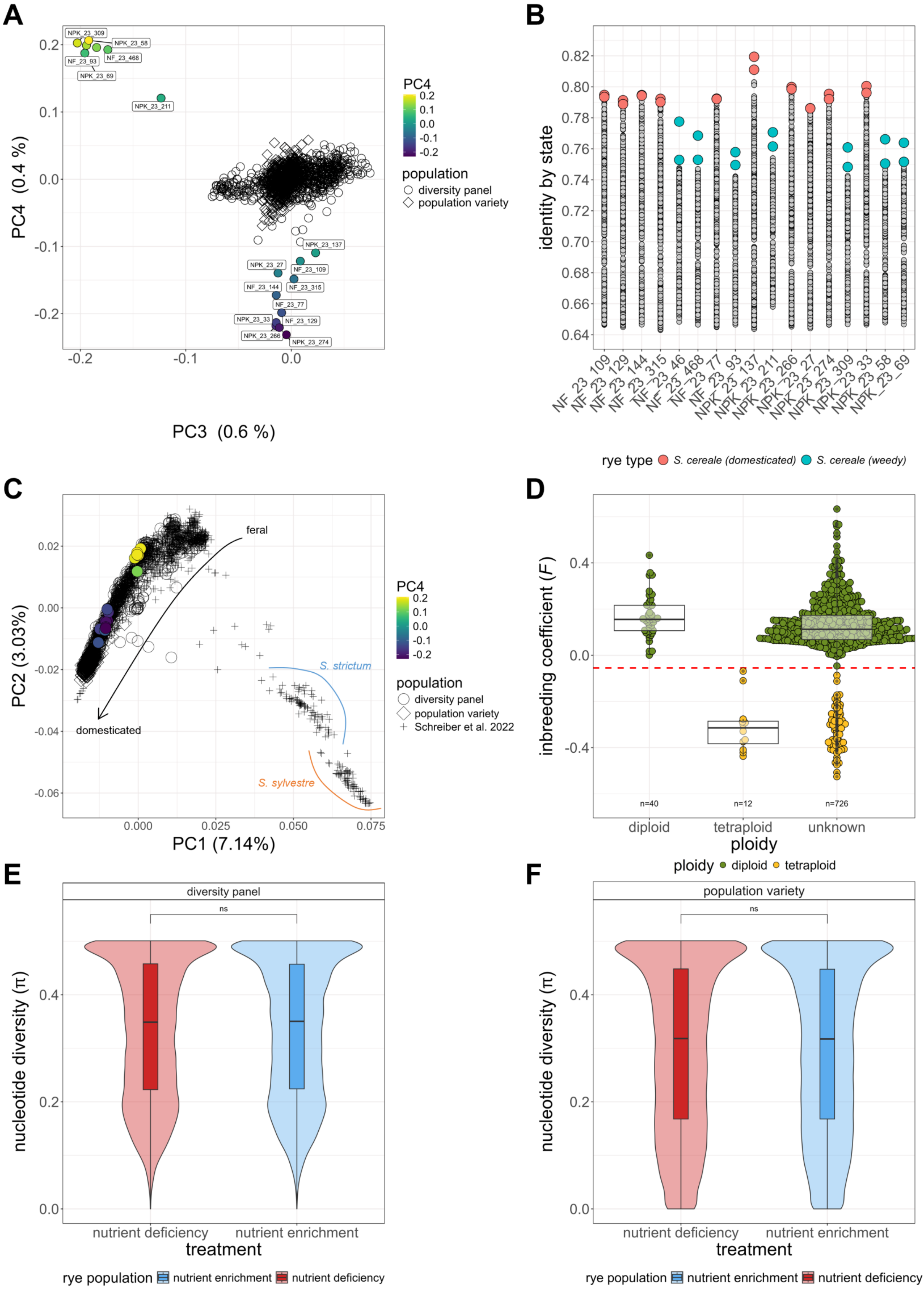

A
NPK_23_309
NPK_23_58
NF_23_468
NF_23_93
NPK_23_69
NPK_23_211
NPK_23_137
NPK_23_27
NF_23_109
NF_23_144
NF_23_315
NF_23_77
NPK_23_33
NF_23_129
NPK_23_266
NPK_23_274
PC4 (0.4 %)
PC3 (0.6 %)
PC4
population
diversity panel
population variety
B
identity by state
NF_23_109
NF_23_129
NF_23_144
NF_23_315
NF_23_46
NF_23_468
NF_23_77
NF_23_93
NPK_23_137
NPK_23_211
NPK_23_266
NPK_23_27
NPK_23_274
NPK_23_309
NPK_23_33
NPK_23_58
NPK_23_69
rye type
S. cereale (domesticated)
S. cereale (weedy)
C
feral
domesticated
S. strictum
S. sylvestre
PC2 (3.03%)
PC1 (7.14%)
PC4
population
diversity panel
population variety
Schreiber et al. 2022
D
inbreeding coefficient (F)
n=40
n=12
n=726
diploid
tetraploid
unknown
ploidy
ploidy
diploid
tetraploid
E
diversity panel
ns
nucleotide diversity (π)
nutrient deficiency
nutrient enrichment
treatment
rye population
nutrient enrichment
nutrient deficiency
F
population variety
ns
nucleotide diversity (π)
nutrient deficiency
nutrient enrichment
treatment
rye population
nutrient enrichment
nutrient deficiency

Supplementary Fig. S2: Identification of origin of rye lines of diversity panel clustering in PC3-PC4. **(A)** PCA Plot of PC3 and PC4 with rye outlier lines separating from main cluster sample names indicated by labels and coloured according to PC4 values, circles and diamonds represent individuals of diversity panel and population variety, respectively. **(B)** Identity by state calculation of rye outlier lines with genotypes of Schreiber et al. 2022; the top two lines with highest identity are highlighted in coral for *S. cereale* (domesticated) and turquoise for *S. cereale* (weedy). **(C)** Joint PCA plot (PC1 vs. PC2) of the Schreiber et al. (2022) rye population, the diversity panel, and the population variety. The gradient of genetic divergence from feral to domesticated rye is indicated by an arrow. *S. sylvestre* (orange) and *S. strictum* (blue) are marked. Rye outlier lines are again coloured according to PC4 values. **(D)** Ploidy estimation of diversity panel genotypes by comparing inbreeding coefficients to diploid (green) and tetraploid (yellow) genotypes from Waesch et al. 2025*b* based on flow cytometry. The tetraploidy threshold is indicated with dashed, red horizontal line at $F \leq -0.05$. **(E–F)** Comparisons of nucleotide diversity between rye lines grown under nutrient enrichment (blue) and nutrient deficiency conditions (red) for diversity panel **(E)** and population variety **(F)** show no significant difference between treatments. Statistical significance was assessed using the Mann–Whitney U Test with Bonferroni correction (ns = not significant).

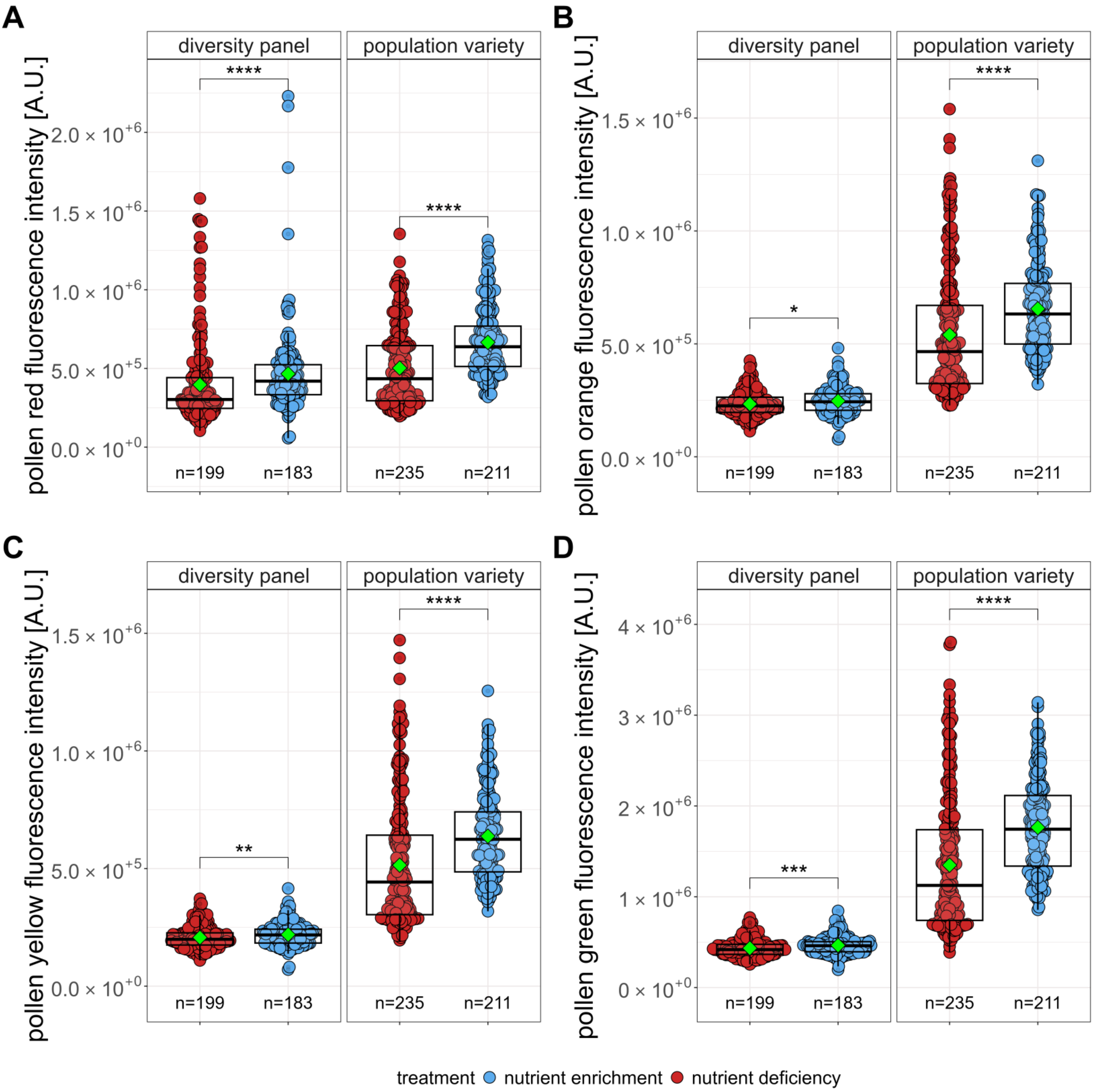

Supplementary Fig. S3: Pollen fluorescence intensity is significantly higher under nutrient enriched conditions for both rye populations. Boxplots for red **(A)**, orange **(B)**, yellow **(C)** and **(D)** green pollen fluorescence intensity comparing nutrient enrichment with nutrient deficiency conditions in population variety and diversity panel; Mann–Whitney U Test with Bonferroni correction applied; ns = not significant, * $P < 0.05$, ** $P < 0.01$ *** $P < 0.001$, **** $P < 0.0001$; color code represents soil nutrient treatment (blue: nutrient enrichment; red: nutrient deficiency), and green diamonds indicate mean values; A.U. = arbitrary units.

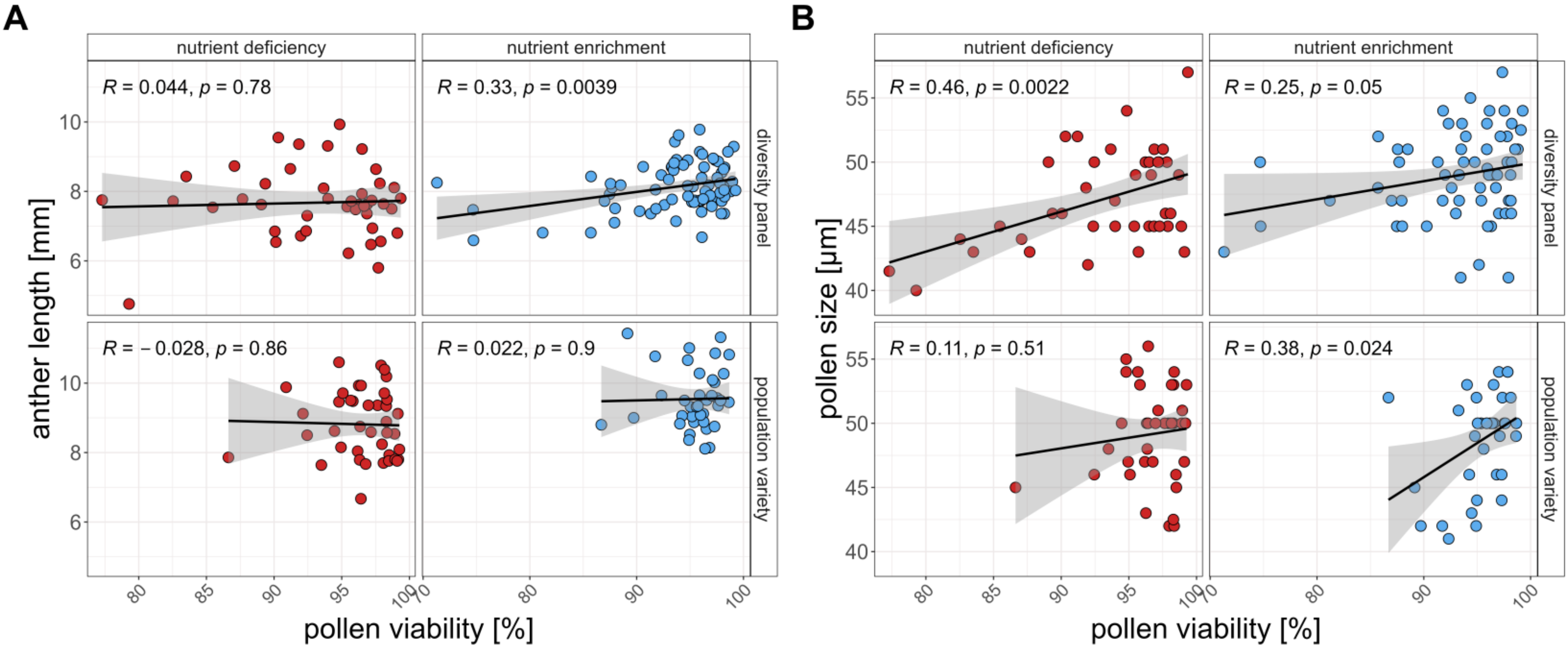

Supplementary Fig. S4: Correlations of pollen viability with anther length **(A)** and pollen size **(B)** under nutrient deficiency and enrichment in rye diversity panel and population variety. Color code indicates soil nutrient treatment (blue: nutrient enrichment, red: nutrient deficiency); Pearson correlation coefficients with *P*-values indicate strength of relationship.

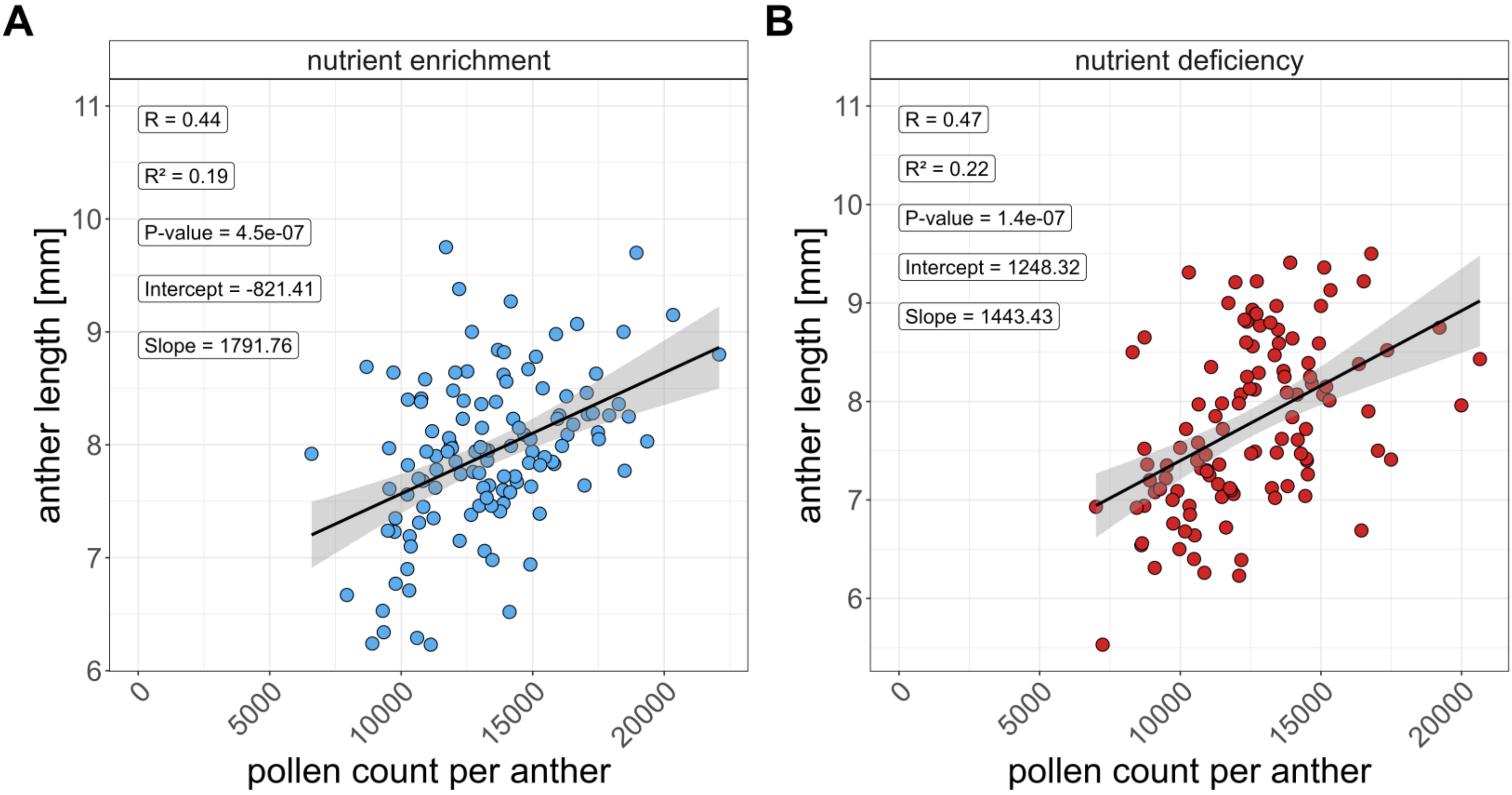

Supplementary Fig. S5: Linear regression model for anther length and pollen count in the diversity panel for nutrient enrichment **(A)** and nutrient deficiency treatment **(B)**.
